# Investigation of Mitochondrial Related Variants in a Cerebral Small Vessel Disease Cohort

**DOI:** 10.1007/s12035-022-02914-3

**Published:** 2022-06-14

**Authors:** P. J. Dunn, N. R. Harvey, N. Maksemous, R. A. Smith, H. G. Sutherland, L. M. Haupt, L. R. Griffiths

**Affiliations:** 1 Centre for Genomics and Personalised Health, Genomics Research Centre, School of Biomedical Sciences, Queensland University of Technology (QUT), 60 Musk Ave., Kelvin Grove, Queensland 4059 Australia; 2 Health Sciences and Medicine Faculty, Bond University, 14 University Drive, Robina, Queensland Australia; 3 Department of Medical and Molecular Genetics, Guy’s Hospital, Kings College London, London, SE1 9RT England

**Keywords:** Mitochondria, Whole exome sequencing, Cerebral small vessel disease, Nuclear-encoded mitochondrial proteins, Encephalopathy, Alzheimer’s disease

## Abstract

Monogenic forms of cerebral small vessel disease (CSVD) can be caused by both variants in nuclear DNA and mitochondrial DNA (mtDNA). Mitochondrial encephalopathy, lactic acidosis, and stroke-like episodes (MELAS) is known to have a phenotype similar to Cerebral Autosomal Dominant Arteriopathy with Sub-cortical Infarcts and Leukoencephalopathy (CADASIL), and can be caused by variants in the mitochondrial genome and in several nuclear-encoded mitochondrial protein (NEMP) genes. The aim of this study was to screen for variants in the mitochondrial genome and NEMP genes in a *NOTCH3*-negative CADASIL cohort, to identify a potential link between mitochondrial dysfunction and CSVD pathology. Whole exome sequencing was performed for 50 patients with CADASIL-like symptomology on the Ion Torrent system. Mitochondrial sequencing was performed using an in-house designed protocol with sequencing run on the Ion GeneStudio S5 Plus (S5 +). NEMP genes and mitochondrial sequencing data were examined for rare (MAF < 0.001), non-synonymous variants that were predicted to have a deleterious effect on the protein. We identified 29 candidate NEMP variants that had links to either MELAS-, encephalopathy-, or Alzheimer’s disease–related phenotypes. Based on these changes, variants affecting *POLG*, *MTO1*, *LONP1*, *NDUFAF6*, *NDUFB3*, and *TCIRG1* were thought to play a potential role in CSVD pathology in this cohort. Overall, the exploration of the mitochondrial genome identified a potential role for mitochondrial related proteins and mtDNA variants contributing to CSVD pathologies.

**Supplementary Information:**

The online version contains supplementary material available at 10.1007/s12035-022-02914-3.

## Background

Mitochondria play an important role in energy metabolism, free-radical generation, and apoptosis [1]. Unlike other organelles, mitochondria are encoded and regulated by a specified genetic architecture that includes the mitochondrial genome and nuclear-encoded mitochondrial protein (NEMP) genes. Unlike genomic DNA (gDNA) which is inherited from both parents, mtDNA is maternally inherited and is composed of ~ 16.6 Kbp of DNA in a plasmid/circular structure that encodes 2 rRNAs, 22 tRNAs, and 13 polypeptides that perform structural or enzymatic functions in the mitochondrion, with the remaining mitochondrial components encoded by the ~ 1200 NEMP genes. Mutations within mtDNA that may affect mitochondrial function have previously been associated with neurological disease, drug toxicities, myopathies, and some cancers [2–4]. While mtDNA plays a major role in diseases associated with mitochondria, the NEMP genes can also contribute to all these disease states.

Stroke and dementia pathologies have also been linked to dysfunctional mitochondria. This has been seen through mitochondrial response, excitotoxicity, acidotoxicity, protein misfolding, and inflammatory reactions [5, 6]. A genetic contribution to stroke and dementia pathologies has also been observed. This is evident with monogenic causes of cerebral small vessel disease (CSVD), such as such as Cerebral Autosomal Dominant Arteriopathy with Sub-cortical Infarcts and Leukoencephalopathy (CADASIL), a condition which causes stroke, dementia, migraine, and epilepsy. CADASIL is a monogenic condition caused by cysteine-altering mutations in *NOTCH3*, where altered mitochondrial proliferation, apoptosis, and overall function have been observed [7].

Furthermore, pathogenic variants identified in mtDNA and NEMP genes have also been shown to cause stroke and dementia phenotypes, in particular in conditions such as mitochondrial encephalopathy with lactic acidosis and strokes (MELAS) [MIM # 540000] and myoclonic epilepsy with ragged red fibres (MERRF) [MIM# 545000]. Symptoms in MELAS include stroke-like episodes, diminished intellectual and cognitive functions, and/or headaches; however, it also include seizures, sensorineural hearing loss, diabetes mellitus, cardiomyopathy, and gastrointestinal dysmotility, whereas MERRF symptoms include generalized epilepsy, ataxia, weakness, and dementia [8, 9]. For both conditions, the majority of variants have been identified in specific mitochondrial encoded genes (*MTTL1*, *MTTQ*, *MTTH*, *MTTK*, *MTTC*, *MTTS1*, *MTTND1*, *MTND5*, *MDTN6*, and *MTTS2*); however, variants in *POLG*, which is one of the NEMP genes, have also been identified to cause MELAS [10]. As mitochondrial irregularities are evident in CADASIL-derived cells, mtDNA and NEMP genes were investigated to identify a potential novel cause of disease in patients referred for CADASIL testing, but which had no pathogenic *NOTCH3* variants.

## Methods

### Patient Cohort

Blood samples were collected from clinically referred CADASIL patients (*n* = 50) who had no pathogenic *NOTCH3* variant, but had signs and symptoms indicative of a monogenic CSVD including recurrent stroke and/or vascular dementia with no other risk factors. As these participants were originally referred from neurologists from around Australia, it was assumed that they all had strong clinical characteristics indicative of CADASIL. In cases where little to no clinical information available, further requests from the referring physicians were completed. Participants who still had no clinical information given were assumed to have a “possible” clinical diagnosis of CADASIL by criteria which includes age of onset ≤ 65 years old, family history of stroke and/or dementia, lack of/mild vascular risk factors, MRI which included white matter change [11, 12]. This resulted in a M:F ratio of 1:2.3 within the cohort with a mean age of 52.2 (± 13.1). All patients had approved diagnostic testing for CADASIL with their doctors, and ethical approval for this study was obtained through the QUT HREC along with appropriate consents for the patient cohort (Approval Number 1800000611). Diagnostic testing was performed using the Genomics Research Centre (GRC) custom 5-gene panel where only the *notch receptor 3* (*NOTCH3*) gene was analysed [13].

### Whole Exome Sequencing

We used an initial input of 80 ng of genomic DNA for whole exome sequencing (WES) using Ion AmpliSeq Exome RDY-kits (Carlsbad, CA, USA) for library preparation, according to manufactures’ instructions (MAN0010084). Completed libraries from samples were quantified using an Invitrogen Qubit 3 Fluorometer (Venlo, Netherlands) and combined at equimolar concentration of 100 pM prior to sequencing. Template preparation, enrichment, and chip loading were performed using the Ion P1 Hi-Q Chef Kit (Cat. Number A26433) or Ion 540™ Chef Kit (Cat. Number A30011) and 540 Chips on the ThermoFisher Scientific Ion Chef (Carlsbad, CA, USA) targeted at 200-bp lengths. Sequencing was performed using the Ion Proton and Ion S5 + platforms with sequencing alignment (Hg19) and variant calling completed via the Ion Torrent software (Carlsbad, CA, USA).

### Mitochondrial Sequencing

Mitochondrial sequencing (MitoSeq) was also completed using the Ion Torrent sequencing system based on a protocol developed by the Genomics Research Centre [14]. This work involved performing two separate long-range PCRs (labelled fragment 1 and fragment 2) on each sample to amplify the mitochondrial DNA. Clean-up of the PCR product was completed using the QIAquick PCR clean-up kit (QIAGEN, Melbourne, Australia) and the resulting products were quantified using Bioanalyser 12,000 Chips (Agilent, Melbourne, Australia). Fragments were then diluted to equimolar concentrations of 20 ng/uL and pooled for each sample. Sonication of each sample with the pooled fragments 1 and 2 was completed on 100 ng of input DNA in 51µL of solution. End repair of the sonicated samples was completed using the NEBNext library preparation for Ion Torrent sequencing kit and BIOO scientific (Adelaide, Australia) barcodes were used to label each sample. The E-gel size selection system with 2% agarose gels was used to select for fragments between 150 and 300 bp in size. A final amplification of these fragments using the NEBNext library preparation for Ion Torrent sequencing was completed and final libraries were quantified on using Bioanalyser 1000 DNA kits or Bioanalyser HS DNA kits if there was a lower-than-expected library yield. Completed libraries were then diluted to 26-pM concentration and different barcodes were pooled to be run on Ion 530 chips in conjunction with the Ion Chef system, according to manufacturer instructions. Following template preparation and amplification, the chips were loaded and sequenced on the Ion S5 + .

### Analysis

Extraction of the rare and functional NEMP gene variants was completed through merging the WES variant call format (vcf) files using the bcftools vcf-merge function. VEP was used to annotate the merged vcf and VEP-filter was used to extract the functional and rare NEMP gene variants [15–17]. This involved thresholds of SIFT scores ≤ 0.05, PolyPhen scores of ≥ 0.8, and a global MAF (gnomAD) of < 0.001. Variants were then further annotated using VariantTaster and PredictSNP2 and stratified according to the number of in silico tools which classified the variant as disease-causing/deleterious. Variant databases such as ClinVar and dbSNP were then used to identify if any variants had previously been found to play a role in disease. Gene ontology for each variant was investigated focusing on tissue distribution of mRNA and protein expression. Samples were removed if there was no protein expression identified through the Protein Atlas Database in smooth muscle cells and neurological tissue. Boolean searches based on gene of interest in Google and NCBI PubMed (https://pubmed.ncbi.nlm.nih.gov/) for Vasc*, Neur* Leukodystrophy, encephalopathy, stroke, and dementia were used to further identify candidate variants. Information based on the Boolean searches was used to identify the remaining candidate variants of interest from the mitochondrial genome and the NEMPs which were then confirmed using Sanger sequencing.

For the MitoSeq data, variant annotation identification was completed through MitoMaster software (https://mitomap.org/foswiki/bin/view/MITOMASTER/WebHome) using MitoMAP. Haplogroups for each sample were investigated using HaploGrep2 (https://haplogrep.i-med.ac.at/category/haplogrep2/) [18]. This initial annotation in MitoMAP identifies MitoTIP scores and further annotation of the merged-vcf data was completed using the MSeqDR mvTool [19]. This software identified population allele frequency; disease phenotypes derived from HmtDB, MitoTIP, MSeqDR, and ClinVar; genomic annotations that were derived from VEP; in silico pathogenicity annotations derived from dbNSF and CADD and finally HmtDB. Only variants with a QUAL score > 20, coverage depth (DP) > 10, and a MAF < 0.001 were considered for pathogenic findings. Variants were excluded if they had previously been classified as polymorphic, likely benign, or benign. Remaining variants were then run through the MitIMPACT 3D (http://mitimpact.css-mendel.it/) tool with further annotation using disease phenotypes. Further filtering initially focussed on genes which had previously been identified with MELAS and/or MERRF and then variant that had previously been identified as pathogenic/likely pathogenic. Finally, remaining candidate variants which were not previously identified as causative of stroke and/or dementia disorders were investigated for gene ontology, gene/protein expression, and function.

## Results

### Mitochondrial Sequencing

The MitoSeq data showed good overall coverage with a median coverage > 1000 × . The high coverage depth also showed a high sequence quality generated with most reads showing a mean sequence Phred score > 25. Furthermore, the quality of nucleotides based on the size of the fragments across samples also shows Phred scores > 25 for all samples with reads up to 250 bp. Across all samples, the read lengths were typically between 100 and 250 bp in size, with most samples having a read length distribution ~ 200 bp. The merged vcf file identified a total of 1178 variants with 302 unique variants across the 50 samples. In conjunction with the extracted mitochondrial reads, there were no variants identified in this sequence data that have previously been identified as causative of MELAS, which included the m.3243G > A variant.

The 302 variants identified from the MitoSeq data were filtered according to the MitoMap minor allele frequency, predicted pathogenicity (MitoMaster, HmtDB), and clinical databases (ClinVar, dbSNP, HmtDB, and MitoMaster). From this, it was identified that there were 84 variants with a MAF < 0.01 (Supplementary_Material_1) which included only 27 variants with a MAF < 0.001. Investigation of all mitochondrial variants using MitoMap in silico tools failed to find sufficient evidence that any would be causative of CSVD. Despite this, the HmtDB pathogenicity prediction tool identified 9 homoplasmic variants as pathogenic/likely pathogenic (Table 1). Whilst it is unlikely these variants were causative of CSVD, additional filtering strategies were conducted. This excluded two variants (MT-ATP6 NC_012920.1:m.9055G > A and MT-CYB NC_012920.1:m.15218A > G) which had a MAF > 0.01 and a further four variants (m.4295A > G, m.4640C > A, m.8616G > T, and m.12634A > G) due to a MAF > 0.001. Three variants (MT-ATP6 NC_012920.1:m.8605C > T, MT-ND5 NC_012920.1:m.13468C > A, and MT-ND5 NC_012920.1:m.13651A > T) were predicted as pathogenic through HmtDB and also had a MAF < 0.001. Investigation into heteroplasmy failed to identify any change predicted to have clinical significance.

**Table 1:** MitoSeq variants with a predicted pathogenicity of likely-pathogenic to pathogenic based on the HmtDB pathogenicity tool

Sample	Gene	HGVS_g	AF mitomap	HmtDB pathogenicity	HmtDB disease score
DGR349	***MT-TI***	NC_012920.1:m.4295A > G	0.0019	likely pathogenic, Hmtdb, tRNA	0.5
DGR327	***MT-ND2***	NC_012920.1:m.4640C > A	0.0035	likely_pathogenic	0.696
DGR366	***MT-ATP6***	NC_012920.1:m.8605C > T	0.0001	pathogenic	0.743
DGR353	***MT-ATP6***	NC_012920.1:m.8616G > T	0.0032	likely_pathogenic	0.763
DGR020, DGR339, DGR340, DGR344, DGR349	***MT-ATP6***	NC_012920.1:m.9055G > A	0.042	likely_pathogenic	0.533
DGR324, DGR337	***MT-ND5***	NC_012920.1:m.12634A > G	0.0028	likely_pathogenic	0.62
DGR338	***MT-ND5***	NC_012920.1:m.13468C > A	0	pathogenic	0.72
DGR037	***MT-ND5***	NC_012920.1:m.13651A > T	-	likely_pathogenic	0.671
DGR069, DGR070	***MT-CYB***	NC_012920.1:m.15218A > G	0.0173	likely_pathogenic	0.656

### WES NEMP Investigations—MELAS-Related Conditions

The assessment of NEMP gene variants identified *n* = 82 variants which passed QC parameters and had a benign/tolerated rating in < 2 of the in silico pathogenicity tools used as well as a MAF < 0.001. All variants were heterozygous and amino acid altering. Filtering based on Boolean searches and mRNA and protein expression data obtained from the Protein Atlas (where applicable) identified *n* = 29 gene variants across the 50 samples detected in the NEMP genes potentially associated with CSVD related pathology or causative of conditions which clinically overlap with CADASIL/CSVD symptoms.

Investigations based on these Boolean searches identified three genes which have been linked to MELAS pathology. The first was a heterozygous missense variant in *POLG* ENST00000268124.5 c.2209G > C p.Gly737Arg (rs121918054). This variant was identified and classified as Likely-Pathogenic—Pathogenic in ClinVar (VCV000013513) associated with multiple mtDNA disorders. However, it must be noted that the majority of these disorders were recessive conditions and that this variant was most likely only classified in this way under the presence of a second mutation. There was another heterozygous variant in a gene which has been associated with MELAS-like disease in *FASTKD2* ENST00000236980.6 c.965C > T p.Pro322Leu (rs748507111). However, the MELAS phenotype associated with gene has been identified to have an autosomal recessive inheritance pattern. The third gene identified and associated with MELAS was *MTO1* from which there were *n* = 2 separate heterozygous variants ENST00000370300.4 c.350G > A p.Arg117His (rs763571382) and ENST00000370300.4 c.1505G > A p.Arg502His (rs201544686).

### WES NEMP Investigations—Encephalopathies

There were *n* = 14 heterozygous variants in genes which have previously been identified to cause encephalopathy; however, all but two were in genes where encephalopathy is associated with an autosomal recessive inheritance and/or childhood/neonatal presentation (Supplementary 2). The remaining two heterozygous changes were both identified in *LONP1* (NM_001276480.1 c.2218G > A p.Val740Met and c.1288G > A p.Val430Met) (Table 2) which has previously been identified to cause an autosomal-dominant mitochondrial encephalopathy. While these particular variants have not been previously reported as disease causing, they have previously been identified to contribute to the CSVD-related phenotype in some patients [20].

**Table 2:** Candidate variants identified from NEMP genes which have previously been identified to either cause MELAS or have protein products altered in MELAS patients. This table also includes the most likely candidate variants that are also associated with encephalopathy (LONP1). In silico pathogenicity scores were obtained using VEP annotation and the PredictSNP2 tool and mRNA and protein expression information for smooth muscle and neurological tissues was obtained from GTEx

Sample	DGR345	DGR025	DGR023	DGR349	DGR353	DGR027
Position	chr2: 207,635,989	chr6: 74,176,064	chr6: 74,191,932	chr15: 89,866,691	chr19: 5,692,117	chr19: 5,696,280
Gene Variant	***FASTKD2**** NM_001136193.2* c.965C > T (p.Pro322Leu)	***MTO1**** NM_133645.3* c.350G > A (p.Arg117His)	***MTO1**** NM_133645.3* c.1505G > A (p.Arg502His)	***POLG**** NM_002693.3* c.2209G > C (p.Gly737Arg)	***LONP1**** NM_001276480.1* c.2218G > A (p.Val740Met)	***LONP1**** NM_001276480.1* c.1288G > A (p.Val430Met)
dbSNP	rs748507111	rs763571382	rs201544686	rs121918054	rs367780804	rs147307965
ClinVar Accession	VCV000214352.2	-	VCV000089037.3	VCV000013513.13	-	-
ClinVar Rating	Likely pathogenic	-	VoUS, likely pathogenic, pathogenic	Pathogenic, likely pathogenic	-	-
ClinVar Disease	No condition provided		AR mitochondrial disease	Multiple mtDNA disorders	-	-
OMIM Diseases	Combined oxidative phosphorylation deficiency 44 (AR)	Combined oxidative phosphorylation deficiency 10 (AR)	Combined oxidative phosphorylation deficiency 10 (AR)	Mitochondrial DNA depletion syndrome 4A (Alpers type) (AR)		
				Mitochondrial DNA depletion syndrome 4B (MNGIE type) (AR)		
				Mitochondrial recessive ataxia syndrome (includes SANDO and SCAE) (AR)		
				Progressive external ophthalmoplegia, autosomal dominant 1 (AD)		
				Progressive external ophthalmoplegia, autosomal recessive 1 (AR)		
SIFT	0	0.01	0	0	0	0.01
PolyPhen	0.999	0.88	0.994	0.988	0.997	0.995
PredictSNP2	D 87%	D 87%	D 87%	D 87%	D 87%	D 87%
CADD	D 80%	D 52%	D 84%	D 84%	D 52%	D 53%
DANN	D 70%	D 77%	D 77%	D 77%	D 72%	D 74%
FATHMM	D 69%	D 83%	D 83%	D 80%	D 81%	D 83%
FunSeq2	B 62%	D 61%	D 61%	D 62%	D 62%	D 62%
GWAVA	D 50%	D 51%	?	D 50%	?	?
Smooth muscle/neurological tissues mRNA Expression (Y/N)	" + / + + "	" + + / + + + "	" + + / + + + "	" + + / + + "	" + / + "	" + / + "
Protein Expression Smooth muscle/CNS Tissue	N/A	" + + / + + "	" + + / + + "	" + + / + + "	" + + / + + "	" + + / + + "
Disease Association	Compound heterozygous variant in *FASTKD2* as the underlying cause of AR MELAS-like syndrome [PMID 28499982]	Downregulated expression of *MTO1* has been identified in MELAS [PMID 31195049]	Downregulated expression of *MTO1* has been identified in MELAS [PMID 31195049]	POLG variants cause stroke-like episodes similar to MELAS and MERRF [PMID 23324391]	LONP1 activity is increased in MELAS cell lysates and can cause an AD Encephalopathy Condition	LONP1 activity is increased in MELAS cell lysates and can cause an AD Encephalopathy Condition

### WES NEMP Investigations—Alzheimer’s Disease and Stroke

There were 7 heterozygous variants in genes which have been implicated to play a role in Alzheimer’s disease (AD) pathology either through functional protein studies or via genetic association studies (Table 3). This included *NDUFAF6*, *NDUFB3*, *TCIRG*, and *BCKDK*, which were all found to be associated with AD through either GWAS or meta-analysis studies [21–25]. Additionally, it was identified that HAGH protein levels were significantly higher in blood in AD patients with the characteristic Apoe-ε4 variant; there was increased expression of *KIF1B* in the brains of AD patients and an increase in blood methylation levels within the *COASY* gene [26, 27]. There was also two heterozygous variants identified were in genes which have been linked to play a role in ischaemic stroke, *MFN1*—through nitric oxide induced fission related to early ischaemic stroke events and *PDK1*—thought to play a role in Ca^2+^-derived activation of platelets contributing to platelet aggregation and ischaemic stroke risk.

**Table 3:** Genetic variants in the CADASIL-related CSVD cohort in genes from which studies have previously identified a link between the gene loci/function and Alzheimer’s disease. In silico pathogenicity scores were obtained using VEP annotation and the PredictSNP2 tool and mRNA and protein expression information for smooth muscle and neurological tissues was obtained from GTEx

Sample	DGR021	DGR344	DGR350	DGR344	DGR337	DGR324	DGR343
Position	1: 10,364,067	2: 201,943,669	8: 96,044,300	11: 67,814,943	16: 1,869,181	16: 31,121,102	17: 40,716,866
Gene Variant	***KIF1B*** NM_183416.4 c.2824C > T (p.Arg942Cys)	***NDUFB3*** ENST00000237889.4 c.64 T > C (p.Trp22Arg)	***NDUFAF6*** NM_152416.4 c.275C > T (p.Ala92Val)	***TCIRG1*** ENST00000265686.3 c.1209G > A (p.Met403Ile)	***HAGH*** ENST00000397353.2 c.332C > T (p.Thr111Ile)	***BCKDK*** ENST00000219794.6 c.373G > A (p.Val125Met)	***COASY*** NM_001042529.3 c.1187A > G (p.Tyr396Cys)
dbSNP	rs542546734	rs142609245	rs142147073	rs140191063	rs375108482	rs141282419	rs375845459
ClinVar accession	-	VCV000252575.5	-	VCV000287328.2	-	VCV000197262.2	-
ClinVar rating	-	Likely pathogenic–pathogenic	-	VoUS	-	VoUS	-
ClinVar disease	-	Mitochondrial complex 1 deficiency (AR)	-	AR osteoporosis 1	-	Branched-chain keto acid dehydrogenase kinase deficiency	-
OMIM disease	Charcot-Marie-Tooth disease, Type 2A1 (AD) [Neuroblastoma, susceptibility to, 1] (AD,SMu) Pheochromocytoma (AD)	Mitochondrial complex I deficiency, nuclear type 25 (AR)	Fanconi renotubular syndrome 5 (AR) Mitochondrial complex I deficiency, nuclear type 17 (AR)	Osteopetrosis, autosomal recessive 1 (AR)	[Glyoxalase II deficiency] (AD)	Branched-chain ketoacid dehydrogenase kinase deficiency	Neurodegeneration with brain iron accumulation 6 (AR) Pontocerebellar hypoplasia, type 12 (AR)
SIFT	0	0	Cerebellar hypoplasia/atrophy, epilepsy, and global developmental delay (AR)	0	0	0	0
PolyPhen	0.959	0.998	0.995	0.986	0.997	0.99	1
PredictSNP2	D 87%	D 87%	D 87%	D 87%	D 87%	D 87%	D 87%
CADD	D 52%	D 52%	D 84%	D 52%	D 71%	D 53%	D 53%
DANN	D 77%	D 62%	D 77%	D 68%	D 70%	D 73%	D 70%
FATHMM	D 77%	D 62%	D 67%	D 79%	D 80%	D 73%	D 67%
FunSeq2	D 77%	D 62%	B 62%	D 61%	D 62%	D 62%	D 62%
GWAVA	D 62%	D 51%	D 51%	D 50%	D 51%	?	?
Smooth Muscle/neurological tissues mRNA expression (Y/N)	" + / + "	" + / + "	" + / + "	" + / + "	" + / + + "	" + / + "	" + / + + "
Protein expression smooth muscle/CNS Tissue	" + + / + + "	" + + / + + "	N/A	" + ± "	" + / + + "	"-/ + "	" + / + "
Alzheimer’s Disease association	Increased expression of KIF1B in the brains of AD patients	NDUFB3 identified as part of a cluster associated with oxidative phosphorylation that plays a major role in Alzheimer’s disease	A genetic signal for AD was detected in NDUFAF6. Also causes Leigh Syndrome—a childhood mitochondrial disease	A missense variant in this gene has been associated with early onset Alzheimer’s disease (EOAD)	GWAS identified protein levels significantly higher Apoe e4 AD patients	AD loci identified in a large meta-analysis in BCKDK	Increased methylation in COASY gene from blood analysis in AD

## Discussion

### Mitochondrial Sequencing Investigations

Initial investigations of known disease-causing variants in the MitoSeq data failed to identify any changes that would result in a known CSVD or neurodegeneration condition such as MELAS. Further investigations focussing on the predicted pathogenic and likely pathogenic variants found 9 variants. The m.4295A > G (*MT-TI)* variant, seen in DGR349, was previously been found to be associated with maternal hypertension and maternal sensory hearing loss [28]. Another case study has also found this variant in an individual who suffered an occipital stroke [29]. The MAF of this variant is also > 0.001 across multiple population databases, making it unlikely that it is causative of CSVD. Despite these findings, there is little evidence to suggest a role for this variant in CSVD or neurodegeneration phenotypes.

In DGR327, there was the m.8605C > T variant which affects *MT-ND2*. This gene is one of the seven mitochondrial genes encoding for subunits of NADH dehydrogenase where variants in this gene have previously been associated with Leber optic atrophy, mitochondrial complex 1 deficiency, and Leigh syndrome due to mitochondrial complex deficiency [30, 31]. With an allele frequency of 0.035, it is unlikely that this variant would be causative of a rare monogenic form of CSVD. Furthermore, there is an additional variant at this loci, m.8605C > A, which may indicate that this region.

Three variants were identified across seven samples in the *MT-ATP6* gene (DGR366 m.8605C > T, DGR353 m.8616G > T, and DGR020, DGR339, DGR340, DGR344, and DGR349 m.9055G > A) that were considered likely pathogenic or pathogenic. For m.8616G > T and m.9055G > A, these variants both had MAF > 0.001 and were thus excluded as likely candidate causes for CSVD. While the m.8605C > T variant had a MAF < 0.001, it was also identified that this position is also known to harbour a different variant, m.8605C > A, which may indicate that this position is more prone to variation than current populations databases reveal. For this reason, it is unlikely that it could be considered a pathogenic cause of CSVD. Variants in *MT-ATP6* have been shown to cause a number of symptoms such as ataxia, cognitive dysfunction, neuropathy, seizures, and retinopathy; however, it is most commonly known to cause mitochondrial complex V deficiency, Leigh syndrome, and/or neuropathy, ataxia and retinitis pigmentosa (NARP) [32]. These conditions primarily affect infants and young children, so it is unlikely that homoplasmic variants within this gene are a cause of CSVD pathology.

There were three homoplasmic variants identified as likely pathogenic or pathogenic in *MT-ND5* across four samples (DGR324 and DGR337 m.12634A > G, DGR338 m.13468, and DGR037 m.13651A > T). Heteroplasmic variants in *MT-ND5* have previously been identified in MELAS patients or Leigh syndrome and there have been some theories that this may be a novel hot spot for MELAS causing variants outside of the classical m.3243A > G variant [33–36]. However, none of the variants described in this literature match one that was seen in the CADASIL-related CSVD cohort. Due to the homoplasmic nature of the variants identified, it is likely that the clinical manifestations would not be limited to neurological and neurovascular phenotypes. Despite this, the m.13468C > A and m.13651A > T variants have not previously been identified in the Mitomap database. The m.13468C > A variant has a total incidence of 3.544 × 10^−5^ in gnomAD, while the m.13651A > T transition has not previously been detected in gnomAD.

The final variant of interest was the homoplasmic *MT-CYB* m.15218A > G which was identified in DGR069 and DGR070. Variants in this gene primarily are associated with Leigh syndrome and exercise intolerance; however, an infantile form of MELAS has also been caused by a 4-bp deletion affecting MT-CYB [37, 38]. As these conditions generally affect young children and infants, and there is a lack of evidence to support homoplasmic changes in this gene as a cause of CSVD or MELAS-like condition, it is unlikely that this variant is causative of disease. Furthermore, the m.15218A > G variant has an allele frequency of 0.014, suggesting that it is at too high of a frequency in the general population to be causative of a rare condition. Overall, there is little evidence at this stage to show that the mitochondrial variants on their own are causative of disease; however, there may be some minor contribution that would require larger scale studies to identify.

### NEMP Genes Associated with MELAS or MELAS-Like Phenotypes

*POLG* encodes for the catalytic subunit DNA polymerase-ɣ enzyme that is solely responsible for mtDNA replication by conferring the proofreading activity of the enzyme [39]. Variants in this gene have been found to be associated with a wide range of phenotypes inherited in an autosomal dominant and recessive manner [40, 41]. This is also true for the variant identified in DGR349 (p.Gly737Arg) which has previously been identified and characterised in ClinVar (VCV000013513.13) and was associated with progressive sclerosing poliodystrophy mitochondrial DNA depletion syndrome 4B—MNGIE type (MIM# 613,662), sensory ataxic neuropathy-dysarthria-ophthalmoparesis syndrome (MIM# 607,459), and POLG-related spectrum disorders, seizures, and progressive external ophthalmoplegia with mitochondrial DNA deletions (autosomal recessive). Depletion in the enzyme activity of POLG may result in mtDNA depletion and/or multiple deletions due to the role POLG plays in mtDNA replication and related processes (e.g. proofreading). *POLG* variants have also previously been associated with MELAS and MELAS-like phenotypes. In addition, although a MAF of 7.5 × 10^−4^ with *n* = 211 allele carriers in the gnomAD database is within the threshold for a rare disorder, it is still considered somewhat high. Furthermore, the high clinical heterogeneity associated with *POLG* variants, and as no family studies were completed for DGR343, it is still unclear if this variant is causative or contributing to the CSVD phenotype. Furthermore, whilst the *POLG* Glycine 373 codon is highly conserved and thus computationally predicted as pathogenic through SIFT and PolyPhen2, it is most commonly associated with POLG-related diseases when there is the presence of a second mutation in the gene. Further functional testing would be recommended and further examination of the exome for DGR343 should be completed.

There was also a heterozygous variant identified in *FASTKD2*. This variant has previously been identified as likely pathogenic in ClinVar (VCV000214352.2) as causative of combined oxidative phosphorylation deficiency type 44 (MIM#612,322). However, variants in *FASTKD2* have also been associated with an autosomal-recessive inherited form of MELAS [42]. Despite this association, it is unlikely that the heterozygous *FASTKD2* variant identified in DGR345 is causative of a MELAS phenotype; however, further study into the effect this variant has on *FASTKD2* function may identify a plausible role for this gene and its variants in CSVD.

Finally, two individuals (DGR023 and DGR025) were identified with separate variants in *MTO1.* The first variant identified in DGR023 results in a p.Arg502His variant (rs201544686) that has previously been cited as causing an autosomal recessive mitochondrial disease in ClinVar (VCV000089037.3). The second variant causes a heterozygous missense change that affects p.Arg117His (rs763571382), which has not been previously classified in ClinVar. Variants in *MTO1* are recognised in OMIM as causing combined oxidation phosphorylation deficiency 10 (MIM# 614,702). What is interesting is that *MTO1* encodes a protein involved in tRNA modification and protein synthesis. In MELAS, the two variants which have been estimated to cause ~ 90% of cases are m.3243G > A (80% of cases) and m.3271 T > C (~ 10% cases) both affect the Mitochondria tRNA Leucine 1 gene (*MT-TL1)* [43, 44]. Furthermore, MELAS patients with the m.3243G > A variant have been found to have a decreased expression of *MTO1* due to the inducing miR-9/9* [45, 46]. The proposed mechanism in MELAS that causes this induction of miR-9/9* and subsequent downregulation of MTO1 relates to oxidative stress and increased levels of intracellular Ca^2+^ and reactive oxygen species (ROS). It would be interesting to investigate the cellular effects caused by the variants in DGR023 and DGR025 to see if this correlates with a decreased expression or activity of MTO1 resulting in a phenotype mimicking MELAS or related to CSVD.

### NEMP Genes Associated with Encephalopathy

There were *n* = 15 variants identified across 12 genes previously associated with encephalopathy. This was the most prevalent neurological symptom identified through the Boolean searches; however, further investigation of the literature found that *n* = 11/12 genes were only linked to encephalopathy through biallelic loss or compound heterozygous variants to have an effect, which caused severe conditions often detected in infancy. Despite this, it is an interesting link as encephalopathy is a common symptom of CSVD and the aetiology of this may be related to mitochondrial dysfunction.

The remaining variants were both identified in *LONP1* (DGR027 p.Val430Met; and DGR353 p.740Met). Interestingly, a recent manuscript identified a de novo dominant variant that causes a mitochondrial encephalopathy with neonatal seizures and death in a 1 year old based on a different variant in *LONP1* [20]. Typically, biallelic variants in *LONP1* cause cerebral, ocular, dental, auricular, and skeletal anomalies (CODAS) syndrome (MIM#600,373), a neonatal multisystem disorder where variants are located in the ATP-dependent protease LON-binding domain. These variants are thought to dramatically increase proteolytic activity of LONP1 [47]. The proteolytic activity of LONP1 is thought to play a role in modulating the abundances of proteins involved in processes relating to environmental cues [20, 48](Zurita Rendón & Shoubridge Eric, 2018). The variants identified in this CSVD cohort affect a different region of the gene and are outside of the key regulatory domains for this protein. Although located outside the functional domains of the protein, these variants may still contribute to the pathogenicity of CSVDs, particularly as these disorders typically manifest later in life. Furthermore, it could be theorised that heterozygous variants in *LONP1* may result in a cumulative effect that causes a later-in-life encephalopathy/cognitive decline [20].

### NEMP Genes Associated with Alzheimer’s Disease Pathology

There were 8 variants identified where the genes were found to be either associated with AD through genetic studies, or the gene products have been shown to behave differently in AD patients. The first gene variant which had an association with AD was the *KIF1B* p.Arg942Cys (rs542546734) variant. KIF1B was identified as upregulated in the brains of AD individual’s post-mortem, from which it was theorised that it may be playing a role not yet defined in AD [26]. Despite the reported expression change, there is still limited evidence to suggest a role of *KIF1B* in AD pathology and as such it is unclear if the variant detected in DGR021 is causative of CSVD. Furthermore, variants in *KIF1B* are documented as causing autosomal dominant inherited disorders including Charcot-Marie Tooth Disease type 2A1 (MIM# 118,210) and pheocychromatoma (MIM# 171,300). Based on other well-established links to disease in an autosomal dominant manner, it is unlikely that variants in *KIF1B* would be a novel candidate gene for CADASIL-related CSVD pathology.

Variants were also identified in DGR026 for *OGDH* p.Arg81Cys and DGR337 *HAGH* p.Thr111Ile, which have both been identified in studies showing that there are gene expression changes detected in AD patients [49, 50]. *OGDH* has been identified to be overexpressed and linked with a protective effect against externally added ROS [49]. Similarly, a GWAS identified a link between elevated levels of HAGH protein in *APOE* ε4 carrier patients [50]. It is unclear and unlikely that the variant detected in DGR337 also causes increased plasma levels of HAGH, as they do not carry the *APOE* ε4/ε4 genotype. As the change in HAGH expression was seen in *APOE* ε4 carrier patients compared to controls, we also cannot rule out that this was a result of an epistatic mechanism and as such a variant in *HAGH* by itself would not be sufficient to contribute to pathology.

Whilst changes in protein and gene expression have been identified in *KIF1B*, *HAGH*, and *OGDH*, association studies have also identified AD loci in *NDUFB3*, *NDUFAF6*, *TCIRG1*, and *BCKDK* [21, 23–25]. There is increasing evidence to support the association of these NEMP genes and AD and it has led to theories of the pathological mechanisms that link AD pathology with disrupted mitochondrial structure and function [6].

DGR344 was found to have a heterozygous *NDUFB3* p.Trp22Arg variant. Interestingly, this variant has previously been found to cause Mitochondrial complex 1 deficiency (MIM 618,246) when it is inherited in a homozygous fashion [51, 52]. *NDUFB3* encodes for an accessory subunit of the oxidative phosphorylation (OXPHOS) complex 1 which is the first enzyme in the electron transport chain in the mitochondria. A reduced expression of OXPHOS protein subunits has been identified and replicated in the blood of AD patients exhibiting mild cognitive impairment which has led to a theory that an imbalance in nuclear and mitochondrial-encoded OXPHOS transcripts may drive a negative feedback loop that reduces OXPHOS efficiency and increases neuronal damage by ROS [53, 54]. As OXPHOS efficiency is impaired in AD patients, further investigation of heterozygous carriers of this *NDUFB3* variant needs to be completed before it could be considered as a candidate gene causative of an AD/CSVD phenotype.

DGR350 had a heterozygous variant in *NDUFAF6* (p.Ala92Val), a gene which was recently identified as a novel locus associated with AD in a meta-analysis focussing on AD and vascular dementia patients [21]. The locus (*NDUFAF6*-rs10098778) identified by Moreno-Grau et al. (2019) is also in high linkage disequilibrium to another marker in the same gene *NDUFAF6-*rs4735340 which was the top signal in a separate meta-analysis which focussed on identifying novel risk loci in late-onset Alzheimer’s disease (LOAD) [55]. The *NDUFAF6* p.Ala92Val variant that was detected in DGR350 has not been classified in ClinVar despite being previously identified by dbSNP and to date there are no functional studies completed on the gene for this position. Albeit, there have been studies that have found that biallelic variants in this gene cause autosomal recessive Leigh syndrome and is associated with mitochondrial complex I deficiency [22]. While Leigh syndrome is a progressive neurodegenerative disease, typically presenting in the first year of life, there is currently no link suggested between this condition and CSVD or AD pathology [56]. Based on this, it is unclear whether variants in *NDUFAF6* may be causing the CSVD phenotype in DGR350 and further studies into heterozygous changes in this gene may be necessary to identify any late-onset related conditions associated with this gene.

There was a heterozygous *TCIRG1* p. Met403Ile variant identified in DGR344. Interestingly, *TCIRG1* has a separate missense variant (*TCIRG1* 11:67,810,477:C > T) that was identified to segregate in 3 separate families with early onset Alzheimer’s disease (EOAD) [24]. TCIRG1 is a component of the a3 subunit of H + -ATPase complex and is located within the lysosomes, where it is critical for the acidification of vacuoles which removes debris via the endolysosomal pathway. Interestingly, lysosomal function has also been theorised to aid in the disposal of the *NOTCH3* extracellular domain (NECD) in wild-type and CADASIL patients [57]. In CADASIL, this may affect disease pathogenesis with a recent study suggesting that a dysfunction in the autophagy-lysosomal pathway may be a key component to the molecular mechanisms of CADASIL [58]. The *TCIRG1* amino acid change in DGR344 (p.Met403Val) is two amino acids upstream from a vacuolar domain in the protein, and it may be that this variant disrupts the helical domain and alters TCIRG1 protein function.

The final variant identified was a heterozygous *COASY* p.Tyr396Cys (rs375845459) variant. This gene was previously identified as associated with AD through increased blood methylation profiles and reported as a potential biomarker for early diagnosis [27]. *COASY* encodes for coenzyme A (CoA) synthase and performs a key role in the biosynthesis of CoA from pantothenic acid [59–61]. It is primarily present in the mitochondrial matrix and variants within the gene are found to alter enzyme activity [27]. Homozygous variants within *COASY* have been associated with neurodegeneration due to iron accumulation and a SNP within exon 4 (rs598126) identified as a risk factor of EOAD in females with down syndrome [62, 63]. The variant in DGR343 is located within the PFAM dephosphorylation-CoA kinase domain and impact the functional activity of the enzyme. *COASY* has been reported to code for three transcript variants which result in tissue-specific isoforms (the alpha enzyme is ubiquitously expressed and the beta transcript is primarily in the brain in humans) making elucidating the functional consequence of variants within the gene difficult, particularly in neurodegenerative disorders [64].

## Conclusion

Overall, this study identified 29 genetic variants in NEMP genes which have previously been implicated as playing a role in CSVD-related diseases or phenotypes. Mitochondrial genome variant screening did not identify any known cause of disease in this cohort, including the m.3243A > G^Leu^ variant causative of MELAS. Extraction of the mitochondrial genome from the Ion AmpliSeq WES was unsuccessful in obtaining sufficient coverage depth across the mitochondrial gnome; however, the MitoSeq was able to mitigate this. MitoSeq was successful in generating a high sequencing depth and allowing for high-quality investigation of mitochondrial variants detected, despite no causative variants being identified. Extraction of the NEMP genes from WES data was able to identify and discover variants which may cause dysfunction in mitochondria and that have previously been associated with CSVD and related neurodegenerative disorders. Most of these genes are associated with severe autosomal recessive conditions and it is unlikely that many are causative of CSVD in this cohort, though there is evidence that variants identified in *POLG*, *MTO1*, *LONP1*, *NDUFAF6*, *NDUFB3*, and *TCIRG1* may be novel causes of CSVD or contributing to a neurodegenerative phenotype. The impact of these genes in CSVD and neurodegeneration should be further investigated.

## Supplementary Information

Below is the link to the electronic supplementary material.

Supplementary file1 (DOCX 36 KB)

Supplementary file2 (DOCX 19.3 KB)

## Data Availability

All data relevant for this study is included within this manuscript or has been submitted to relevant databases. This includes genetic variant information which has been submitted to ClinVar and dbSNP. Due to the medical nature and ethical agreements for use of samples, any further information may be made available on request.

## References

[1] Murphy E (2016). Mitochondrial function, biology, and role in disease. Circ Res.

[2] Taylor RW, Turnbull DM (2005). Mitochondrial DNA mutations in human disease. Nat Rev Genet.

[3] Tuppen HA (2010). Mitochondrial DNA mutations and human disease. Biochim Biophys Acta.

[4] Alston CL (2017). The genetics and pathology of mitochondrial disease. J Pathol.

[5] Liu F (2018). Mitochondria in ischemic stroke: new insight and implications. Aging Dis.

[6] Wang W (2020). Mitochondria dysfunction in the pathogenesis of Alzheimer’s disease: recent advances. Mol Neurodegener.

[7] Viitanen M (2013). Experimental studies of mitochondrial function in CADASIL vascular smooth muscle cells. Exp Cell Res.

[8] Goto Y-I, Nonaka I, Horai S (1990). A mutation in the tRNALeu(UUR) gene associated with the MELAS subgroup of mitochondrial encephalomyopathies. Nature.

[9] Pavlakis SG (1984). Mitochondrial myopathy, encephalopathy, lactic acidosis, and strokelike episodes: a distinctive clinical syndrome. Ann Neurol.

[10] Cheldi A (2013). POLG1 mutations and stroke like episodes: a distinct clinical entity rather than an atypical MELAS syndrome. BMC Neurol.

[11] Davous P (1998). CADASIL: a review with proposed diagnostic criteria. Eur J Neurol.

[12] Mizuta I (2017). New diagnostic criteria for cerebral autosomal dominant arteriopathy with subcortical infarcts and leukocencephalopathy in Japan. J Neurol Sci.

[13] Maksemous N (2016). Targeted next generation sequencing identifies novel NOTCH3 gene mutations in CADASIL diagnostics patients. Hum Genomics.

[14] Harvey NR (2019). Ion torrent high throughput mitochondrial genome sequencing (HTMGS). PLoS ONE.

[15] Calvo SE, Clauser KR, Mootha VK (2016). MitoCarta2.0: an updated inventory of mammalian mitochondrial proteins. Nucleic Acids Res.

[16] Falk MJ (2012). Mitochondrial disease genetic diagnostics: optimized whole-exome analysis for all MitoCarta nuclear genes and the mitochondrial genome. Discov Med.

[17] Pagliarini DJ (2008). A mitochondrial protein compendium elucidates complex I disease biology. Cell.

[18] Weissensteiner H (2016). HaploGrep 2: mitochondrial haplogroup classification in the era of high-throughput sequencing. Nucleic Acids Res.

[19] Sonney S (2017). Predicting the pathogenicity of novel variants in mitochondrial tRNA with MitoTIP. PLoS Comput Biol.

[20] Besse A (2020). LONP1 de novo dominant mutation causes mitochondrial encephalopathy with loss of LONP1 chaperone activity and excessive LONP1 proteolytic activity. Mitochondrion.

[21] Moreno-Grau S (2019). Genome-wide association analysis of dementia and its clinical endophenotypes reveal novel loci associated with Alzheimer’s disease and three causality networks: the GR@ACE project. Alzheimers Dement.

[22] Catania A (2018). Compound heterozygous missense and deep intronic variants in NDUFAF6 unraveled by exome sequencing and mRNA analysis. J Hum Genet.

[23] Zhang L (2015). Potential hippocampal genes and pathways involved in Alzheimer’s disease: a bioinformatic analysis. Genet Mol Res.

[24] Kunkle BW (2017). Early-onset Alzheimer disease and candidate risk genes involved in endolysosomal transport. JAMA Neurol.

[25] Marioni RE (2018). *GWAS on family history of Alzheimer*’*s disease*. Transl Psychiatry.

[26] Hares K (2017). Overexpression of kinesin superfamily motor proteins in Alzheimer’s disease. J Alzheimers Dis.

[27] Kobayashi N (2020). Increased blood COASY DNA methylation levels a potential biomarker for early pathology of Alzheimer’s disease. Sci Rep.

[28] Li Z (2008). Maternally inherited hypertension is associated with the mitochondrial tRNA(Ile) A4295G mutation in a Chinese family. Biochem Biophys Res Commun.

[29] Finnilä S, Hassinen IE, Majamaa K (2001). Phylogenetic analysis of mitochondrial DNA in patients with an occipital stroke: evaluation of mutations by using sequence data on the entire coding region. Mutat Res/Mutat Res Genom.

[30] Hinttala R (2006). Analysis of mitochondrial DNA sequences in patients with isolated or combined oxidative phosphorylation system deficiency. J Med Genet.

[31] Johns DR, Berman J (1991). Alternative, simultaneous complex I mitochondrial DNA mutations in Leber’s hereditary optic neuropathy. Biochem Biophys Res Commun.

[32] Stendel C (2020). Delineating <em>MT-ATP6</em>-associated disease. Neurol Genet.

[33] Liolitsa D (2003). Is the mitochondrial complex I ND5 gene a hot-spot for MELAS causing mutations?. Ann Neurol.

[34] Dickerson BC (2005). Case records of the Massachusetts General Hospital. Case 36–2005. A 61-year-old woman with seizure, disturbed gait, and altered mental status. N Engl J Med.

[35] Shanske S (2008). The G13513A mutation in the ND5 gene of mitochondrial DNA as a common cause of MELAS or Leigh syndrome: evidence from 12 cases. Arch Neurol.

[36] Panadés-de Oliveira L (2020). A novel mutation in the mitochondrial MT-ND5 gene in a family with MELAS. The relevance of genetic analysis on targeted tissues. Mitochondrion.

[37] De Coo IF (1999). A 4-base pair deletion in the mitochondrial cytochrome b gene associated with parkinsonism/MELAS overlap syndrome. Ann Neurol.

[38] Rana M (2000). An out-of-frame cytochrome b gene deletion from a patient with parkinsonism is associated with impaired complex III assembly and an increase in free radical production. Ann Neurol.

[39] Lamantea E (2002). Mutations of mitochondrial DNA polymerase γA are a frequent cause of autosomal dominant or recessive progressive external ophthalmoplegia. Ann Neurol.

[40] Horvath R (2006). Phenotypic spectrum associated with mutations of the mitochondrial polymerase gamma gene. Brain.

[41] Wong L-JC (2008). Molecular and clinical genetics of mitochondrial diseases due to POLG mutations. Hum Mutat.

[42] Yoo DH (2017). Identification of FASTKD2 compound heterozygous mutations as the underlying cause of autosomal recessive MELAS-like syndrome. Mitochondrion.

[43] Ikeda T (2018). Mitochondrial DNA 3243A>T mutation in a patient with MELAS syndrome. Human Genome Variation.

[44] Niedermayr K (2018). Mitochondrial DNA mutation “m.3243A>G”-Heterogeneous clinical picture for cardiologists (“m.3243A>G”: A phenotypic chameleon). Congenit Heart Dis.

[45] Meseguer S (1866). (2019) The MELAS mutation m.3243A>G alters the expression of mitochondrial tRNA fragments. Biochim Biophys Acta (BBA)- Mol Cell Res..

[46] Meseguer S (2017). microRNA-mediated differential expression of TRMU, GTPBP3 and MTO1 in cell models of mitochondrial-DNA diseases. Sci Rep.

[47] Strauss, Kevin A (2015). CODAS Syndrome is associated with mutations of LONP1, encoding mitochondrial AAA+ Lon protease. Am J Hum Genet.

[48] Zurita Rendón O, Shoubridge Eric A (2018). LONP1 is required for maturation of a subset of mitochondrial proteins, and its loss elicits an integrated stress response. Mol Cell Biol.

[49] Chen H (2016). Reductions in the mitochondrial enzyme α-ketoglutarate dehydrogenase complex in neurodegenerative disease - beneficial or detrimental?. J Neurochem.

[50] Ahmad S (2020). CDH6 and HAGH protein levels in plasma associate with Alzheimer’s disease in APOE ε4 carriers. Sci Rep.

[51] Haack TB (2012). Molecular diagnosis in mitochondrial complex I deficiency using exome sequencing. J Med Genet.

[52] Alston CL (2016). A recurrent mitochondrial p.Trp22Arg NDUFB3 variant causes a distinctive facial appearance, short stature and a mild biochemical and clinical phenotype. J Med Genet.

[53] Lunnon K (2017). Mitochondrial genes are altered in blood early in Alzheimer’s disease. Neurobiol Aging.

[54] Lunnon K (2012). Mitochondrial dysfunction and immune activation are detectable in early Alzheimer’s disease blood. J Alzheimers Dis.

[55] Kunkle BW (2019). Genetic meta-analysis of diagnosed Alzheimer’s disease identifies new risk loci and implicates Aβ, tau, immunity and lipid processing. Nat Genet.

[56] Dahl HH (1998). Getting to the nucleus of mitochondrial disorders: identification of respiratory chain-enzyme genes causing Leigh syndrome. Am J Hum Genet.

[57] Jia L (2009). Lysosome-dependent degradation of Notch3. Int J Biochem Cell Biol.

[58] Hanemaaijer ES (2018). Autophagy-lysosomal defect in human CADASIL vascular smooth muscle cells. Eur J Cell Biol.

[59] Davaapil H, Tsuchiya Y, Gout I (2014). Signalling functions of coenzyme A and its derivatives in mammalian cells. Biochem Soc Trans.

[60] Brass EP (1994). Overview of coenzyme A metabolism and its role in cellular toxicity. Chem Biol Interact.

[61] Gout I (2018). Coenzyme A, protein CoAlation and redox regulation in mammalian cells. Biochem Soc Trans.

[62] Dusi S (2014). Exome sequence reveals mutations in CoA synthase as a cause of neurodegeneration with brain iron accumulation. Am J Hum Genet.

[63] Lee JH (2012). Polymorphisms in HSD17B1: early onset and increased risk of Alzheimer’s disease in women with Down syndrome. Curr Gerontol Geriatr Res.

[64] Nemazanyy I (2006). Identification of a novel CoA synthase isoform, which is primarily expressed in the brain. Biochem Biophys Res Commun.

