# Supplementary material for: Investigation of Mitochondrial Related Variants in a Cerebral Small Vessel Disease Cohort
Source: Mol Neurobiol. 2022 Jun 14;59(9):5366–78. doi: 10.1007/s12035-022-02914-3 (PMC9395495; doi:10.1007/s12035-022-02914-3)
Supplement: Supplementary file 1 — Supplementary file1 (DOCX 36 KB) [file 12035_2022_2914_MOESM1_ESM.docx]

## Supplementary 1 – MitoSeq variants idENTiFied from MitoMaster – MAF <0.01

| Input | HGVS_g | Mitomap AF | HmtDB Pathogenicity | MitoTip Classification | Mitomap Disease | HmtDB Disease | dbSNP | ClinVar Clinical Significance |
| --- | --- | --- | --- | --- | --- | --- | --- | --- |
| 203>A | NC_012920.1:m.203G>A | 0.0046 | pending classification, Hmtdb | - | - | "breast tumor, normal tissues" | - | - |
| 215>G | NC_012920.1:m.215A>G | 0.0092 | pending classification, Hmtdb | - | - | esophageal cancer | rs372439069 | - |
| 222>T | NC_012920.1:m.222C>T | 0.0006 | - | - | - | - | - | - |
| 242>T | NC_012920.1:m.242C>T | 0.0039 | pending classification, Hmtdb | - | - | POLG/PEO muscle | - | - |
| 257>G | NC_012920.1:m.257A>G | 0.0025 | - | - | - | - | - | - |
| 302>ACCC | NC_012920.1:m.302A>C | 0.0005 | - | - | - | - | - | - |
| 308>CCCT | NC_012920.1:m.308C>T | 0.0005 | - | - | - | - | - | - |
| 319>C | NC_012920.1:m.319T>C | 0.004 | - | - | - | - | - | - |
| 338>T | NC_012920.1:m.338C>T | 0.0012 | - | - | - | - | - | - |
| 344>C | NC_012920.1:m.344T>C | 0 | - | - | - | - | - | - |
| 477>C | NC_012920.1:m.477T>C | 0.0092 | pending classification, Hmtdb | - | - | "AD brains, ovarian tumor" | rs41442247 | - |
| 721>C | NC_012920.1:m.721T>C | 0.0025 | pending classification, Hmtdb | - | Possibly LVNC-associated | - | - | - |
| 1721>T | NC_012920.1:m.1721C>T | 0.0061 | pending classification, Hmtdb | - | - | "acute leukemia platelets, leu | rs200626438 | - |
| 1836>G | NC_012920.1:m.1836A>G | 0.0004 | - | - | - | - | - | Uncertain significance |
| 2120>A | NC_012920.1:m.2120G>A | 0.0004 | - | - | - | - | - | - |
| 2158>C | NC_012920.1:m.2158T>C | 0.004 | pending classification, Hmtdb | - | - | - | rs41349444 | - |
| 2707>G | NC_012920.1:m.2707A>G | 0.001 | - | - | - | - | - | - |
| 2863>C | NC_012920.1:m.2863T>C | 0.0017 | pending classification, Hmtdb | - | - | - | rs28393169 | - |
| 3687>T | NC_012920.1:m.3687C>T | 0.0008 | - | - | - | - | - | - |
| 3826>C | NC_012920.1:m.3826T>C | 0.0013 | - | - | - | - | - | - |
| 3834>A | NC_012920.1:m.3834G>A | 0.0086 | pending classification, Hmtdb | - | - | - | rs372080842 | - |
| 3861>G | NC_012920.1:m.3861A>G | 0.0011 | - | - | - | - | - | - |
| 4107>T | NC_012920.1:m.4107C>T | 0.0005 | - | - | - | - | - | - |
| 4188>G | NC_012920.1:m.4188A>G | 0.0037 | pending classification, Hmtdb | - | - | breast cyst | - | - |
| 4295>G | NC_012920.1:m.4295A>G | 0.0019 | likely pathogenic, Hmtdb, tRNA | possibly pathogenic | MHCM / Maternally inherited hypertension / Maternally inherited deafness | - | rs121434467 | Conflicting interpretations of pathogenicity |
| 4679>C | NC_012920.1:m.4679T>C | 0.0005 | - | - | - | - | - | - |
| 4727>G | NC_012920.1:m.4727A>G | 0.0063 | - | - | - | - | - | - |
| 5225>G | NC_012920.1:m.5225A>G | 0 | - | - | - | - | - | - |
| 5892>C | NC_012920.1:m.5892T>C | 0.0017 | pending classification, Hmtdb | - | - | colonic crypts | - | - |
| 5964>C | NC_012920.1:m.5964T>C | 0.0032 | - | - | - | - | - | - |
| 6164>T | NC_012920.1:m.6164C>T | 0.0024 | - | - | - | - | - | - |
| 6257>A | NC_012920.1:m.6257G>A | 0.0083 | pending classification, Hmtdb | - | - | - | rs2856983 | - |
| 6272>G | NC_012920.1:m.6272A>G | 0.0019 | - | - | - | - | - | - |
| 6716>G | NC_012920.1:m.6716A>G | 0.0006 | - | - | - | - | - | Pathogenic |
| 7202>G | NC_012920.1:m.7202A>G | 0.003 | pending classification, Hmtdb | - | - | - | rs369698068 | - |
| 8149>G | NC_012920.1:m.8149A>G | 0.0079 | pending classification, Hmtdb | - | - | - | - | - |
| 8440>G | NC_012920.1:m.8440A>G | 0.0045 | - | - | - | - | - | - |
| 8605>T | NC_012920.1:m.8605C>T | 0.0001 | pathogenic | - | - | - | - | Uncertain significance |
| 8703>T | NC_012920.1:m.8703C>T | 0.0032 | - | - | - | - | - | - |
| 9254>G | NC_012920.1:m.9254A>G | 0.0082 | - | - | - | - | - | - |
| 9377>G | NC_012920.1:m.9377A>G | 0.0082 | - | - | - | - | rs28380140 | - |
| 9380>A | NC_012920.1:m.9380G>A | 0.0094 | - | - | - | - | - | - |
| 9656>C | NC_012920.1:m.9656T>C | 0.0042 | - | - | - | - | - | - |
| 9770>C | NC_012920.1:m.9770T>C | 0.0011 | - | - | - | - | - | - |
| 9773>T | NC_012920.1:m.9773C>T | 0.0002 | - | - | - | - | - | - |
| 10172>A | NC_012920.1:m.10172G>A | 0.0084 | - | - | - | - | - | - |
| 10583>G | NC_012920.1:m.10583A>G | 0.0009 | pending classification, Hmtdb | - | - | - | - | - |
| 10619>T | NC_012920.1:m.10619C>T | 0.0015 | - | - | - | - | - | - |
| 10685>A | NC_012920.1:m.10685G>A | 0.0054 | pending classification, Hmtdb | - | - | - | - | - |
| 10858>C | NC_012920.1:m.10858T>C | 0.0003 | - | - | - | - | - | - |
| 11353>C | NC_012920.1:m.11353T>C | 0.0028 | - | - | - | - | - | - |
| 11485>C | NC_012920.1:m.11485T>C | 0.008 | pending classification, Hmtdb | - | - | - | rs28529320 | - |
| 11653>G | NC_012920.1:m.11653A>G | 0.005 | - | - | - | - | - | - |
| 11662>C | NC_012920.1:m.11662T>C | 0.0003 | - | - | - | - | - | - |
| 11840>T | NC_012920.1:m.11840C>T | 0.004 | - | - | - | - | rs28550734 | - |
| 11929>C | NC_012920.1:m.11929T>C | 0.0022 | - | - | - | - | - | - |
| 12699>G | NC_012920.1:m.12699A>G | 0.0003 | - | - | - | - | - | - |
| 12783>C | NC_012920.1:m.12783T>C | 0.0001 | - | - | - | - | - | - |
| 13098>G | NC_012920.1:m.13098A>G | 0.0004 | - | - | - | - | - | - |
| 13281>C | NC_012920.1:m.13281T>C | 0.0035 | - | - | - | - | - | - |
| 13468>A | NC_012920.1:m.13468C>A | 0 | pathogenic | - | - | - | - | Uncertain significance |
| 13707>A | NC_012920.1:m.13707G>A | 0.0005 | pending classification, Hmtdb | - | - | - | - | - |
| 13740>C | NC_012920.1:m.13740T>C | 0.0041 | - | - | - | - | rs28630861 | - |
| 13743>C | NC_012920.1:m.13743T>C | 0.0044 | pending classification, Hmtdb | - | - | - | - | - |
| 13830>C | NC_012920.1:m.13830T>C | 0.002 | pending classification, Hmtdb | - | - | - | - | - |
| 14139>G | NC_012920.1:m.14139A>G | 0.0063 | - | - | - | - | - | - |
| 14548>G | NC_012920.1:m.14548A>G | 0.0002 | - | - | - | - | - | - |
| 14869>A | NC_012920.1:m.14869G>A | 0.0023 | pending classification, Hmtdb | - | - | breast tumor | - | - |
| 14929>T | NC_012920.1:m.14929C>T | 0.0004 | - | - | - | - | - | - |
| 15253>G | NC_012920.1:m.15253A>G | 0.0006 | - | - | - | - | - | - |
| 15454>C | NC_012920.1:m.15454T>C | 0.0078 | - | - | - | - | - | - |
| 15625>A | NC_012920.1:m.15625C>A | 0.0002 | - | - | - | - | - | - |
| 16114>T | NC_012920.1:m.16114C>T | 0.0023 | - | - | - | - | - | - |
| 16221>T | NC_012920.1:m.16221C>T | 0.0069 | - | - | - | - | rs386829279 | - |
| 16222>T | NC_012920.1:m.16222C>T | 0.008 | pending classification, Hmtdb | - | - | - | rs386829280 | - |
| 16231>C | NC_012920.1:m.16231T>C | 0.009 | pending classification, Hmtdb | - | - | - | - | - |
| 16245>T | NC_012920.1:m.16245C>T | 0.0039 | pending classification, Hmtdb | - | - | - | - | - |
| 16258>C | NC_012920.1:m.16258A>C | 0.0021 | pending classification, Hmtdb | - | - | - | - | - |
| 16287>T | NC_012920.1:m.16287C>T | 0.0053 | pending classification, Hmtdb | - | - | - | rs376279087 | - |
| 16299>G | NC_012920.1:m.16299A>G | 0.0034 | - | - | - | - | rs149142060 | - |
| 16323>C | NC_012920.1:m.16323T>C | 0.0001 | - | - | - | - | - | - |
| 16344>T | NC_012920.1:m.16344C>T | 0.0055 | pending classification, Hmtdb | - | - | - | rs141667876 | - |
| 16398>A | NC_012920.1:m.16398G>A | 0.0013 | - | - | - | - | - | - |
| 16482>G | NC_012920.1:m.16482A>G | 0.0066 | - | - | - | - | - | - |
