# Supplementary material for: Investigation of Mitochondrial Related Variants in a Cerebral Small Vessel Disease Cohort
Source: Mol Neurobiol. 2022 Jun 14;59(9):5366–78. doi: 10.1007/s12035-022-02914-3 (PMC9395495; doi:10.1007/s12035-022-02914-3)
Supplement: Supplementary file 2 — Supplementary file2 (DOCX 19.3 KB) [file 12035_2022_2914_MOESM2_ESM.docx]

## SUPPLEMENTARY 2 – NEMP gene mutations identified that are associated with encephalopathy – with in silico pathogenicity scores

NEMP genes associated with encephalopathy and their predicted in silico pathogenicity scores. D = damaging/deleterious. B = benign/tolerated. ? = unknown impact/score not available. SIFT scores <0.05 are considered pathogenic. PolyPhen scores >0.8 were considered pathogenic

| Sample | Position | Gene Mutation | dbSNP | SIFT | PolyPhen | PredictSNP2 | CADD | DANN | FATHMM | FunSeq2 | GWAVA |
| --- | --- | --- | --- | --- | --- | --- | --- | --- | --- | --- | --- |
| DGR020 | chr1: 156563791 | ***GPATCH4*** NM_144772.3 c.782G>A (p.Arg261His) | rs771620267 | 0 | 0.999 | D 87% | D 53% | D 77% | D 81% | D 61% | D 51% |
| DGR345 | chr2: 207012334 | ***NDUFS1*** ENST00000233190.6 c.472C>T (p.Arg158Cys) | rs372460771 | 0 | 0.997 | D 87% | D 84% | D 77% | D 67% | D 62% | ? |
| DGR307 | chr2: 26414367 | ***HADHA*** ENST00000380649.3 c.2131C>A (p.Pro711Thr) | rs138966725 | 0 | 0.997 | D 87% | D 52% | D 70% | D 76% | D 61% | ? |
| DGR331 | chr2: 26427022 | ***HADHA*** ENST00000380649.3 c.1129G>A (p.Ala377Thr) | rs761474139 | 0.03 | 0.985 | D 87% | D 84% | D 77% | D 83% | D 61% | D 51% |
| DGR021 | chr3: 158407990 | ***GFM1*** ENST00000264263.5 c.2005A>G (p.Met669Val) | rs147847472 | 0.01 | 0.997 | D 87% | D 51% | D 60% | D 83% | D 61% | D 51% |
| DGR024 | chr5: 125887751 | ***ALDH7A1*** NM_001201377.2 c.1195G>C (p.Glu399Gln) | rs121912707 | 0 | 0.999 | D 87% | D 52% | D 72% | D 83% | D 61% | ? |
| DGR324 | chr6: 80878662 | ***BCKDHB*** ENST00000320393.6 c.548G>C (p.Arg183Pro) | rs79761867 | 0 | 1 | D 87% | D 84% | D 66% | D 83% | D 61% | D 51% |
| DGR023 | chr6: 80910740 | ***BCKDHB*** ENST00000320393.6 c.832G>A (p.Gly278Ser) | rs386834233 | 0.02 | 1 | D 87% | D 84% | D 72% | D 79% | D 61% | D 50% |
| DGR032 | chr10: 74311074 | ***MICU1*** ENST00000361114.5 c.356G>A (p.Arg119Gln) | rs767831720 | 0 | 0.998 | D 87% | D 84% | D 77% | D 79% | B 62% | D 51% |
| DGR339 | chr10: 101486760 | ***COX15*** ENST00000016171.5 c.547C>T (p.Arg183Cys) | rs371107669 | 0.02 | 0.954 | D 87% | D 51% | D 77% | D 62% | D 61% | D 51% |
| DGR075 | chr11: 66638798 | ***PC*** ENST00000355677.3 c.475G>A (p.Ala159Thr) | rs768981875 | 0 | 0.907 | D 87% | D 84% | D 77% | D 65% | D 61% | D 51% |
| DGR338 | chr11: 68530186 | ***CPT1A*** ENST00000265641.5 c.1784G>A (p.Arg595Gln) | rs763226691 | 0.02 | 0.962 | D 87% | D 84% | D 77% | D 72% | D 61% | D 51% |
| DGR353 | chr19: 5692117 | ***LONP1*** *NM_001276480.1* c.2218G>A (p.Val740Met) | rs367780804 | 0 | 0.997 | D 87% | D 52% | D 72% | D 81% | D 62% | ? |
| DGR027 | chr19: 5696280 | ***LONP1*** *NM_001276480.1* c.1288G>A (p.Val430Met) | rs147307965 | 0.01 | 0.995 | D 87% | D 53% | D 74% | D 83% | D 62% | ? |
| DGR342 | chr19: 39976855 | ***TIMM50*** ENST00000314349.4 c.830G>A (p.Arg277His) | rs776988071 | 0 | 0.987 | D 87% | D 84% | D 77% | D 72% | D 62% | D 51% |
